# RBM5-AS1 promotes radioresistance in medulloblastoma through stabilization of SIRT6 protein

**DOI:** 10.1186/s40478-021-01218-2

**Published:** 2021-07-05

**Authors:** Chuanying Zhu, Keke Li, Mawei Jiang, Siyu Chen

**Affiliations:** Department of Oncology, Xin Hua Hospital Affiliated to Shanghai Jiaotong University School of Medicine, Shanghai, 200092 China

**Keywords:** Medulloblastoma, RBM5-AS1, Protein stability, Radioresistance, Stemness

## Abstract

Cancer stem cells (CSCs) contribute to radioresistance in medulloblastoma. Thus, identification of key regulators of medulloblastoma stemness is critical for improving radiotherapy for medulloblastoma. In the present study, we profiled CSC-related long non-coding RNAs (lncRNAs) between radioresistant and parental medulloblastoma cells. The roles of the lncRNA RBM5-AS1 in the stemness and radiosensitivity of medulloblastoma cells were investigated. We found that RBM5-AS1, a novel inducer of medulloblastoma stemness, was significantly upregulated in radioresistant medulloblastoma cells compared to parental cells. Knockdown of RBM5-AS1 diminished the viability and clonogenic survival of both radioresistant and parental medulloblastoma cells after radiation. Silencing of RBM5-AS1 significantly enhanced radiation-induced apoptosis and DNA damage. In vivo studies confirmed that depletion of RBM5-AS1 inhibited tumor growth and increased radiosensitivity in a medulloblastoma xenograft model. In contrast, overexpression of RBM5-AS1 reduced radiation-induced apoptosis and DNA damage in medulloblastoma cells. Mechanistically, RBM5-AS1 interacted with and stabilized sirtuin 6 (SIRT6) protein. Silencing of SIRT6 reduced the stemness and reinforced radiation-induced DNA damage in medulloblastoma cells. Overexpression of SIRT6 rescued medulloblastoma cells from RBM5-AS1 depletion-induced radiosensitization and DNA damage. Overall, we identify RBM5-AS1 as an inducer of stemness and radioresistance in medulloblastoma. Targeting RBM5-AS1 may represent a potential strategy to overcome the resistance to radiotherapy in this malignancy.

**Supplementary Information:**

The online version contains supplementary material available at 10.1186/s40478-021-01218-2.

## Introduction

Medulloblastoma is one of the most common pediatric malignancies, accounting for 15–20% of all tumors of the central nervous system in children [1]. Activation of oncogenic networks including HIPPO-YAP/TAZ and AURORA-A/MYCN pathways has been shown to promote medulloblastoma tumorigenesis and relapse [2]. Multimodal regimens including maximal surgical resection, radiotherapy, and chemotherapy are recommended as a standard treatment for medulloblastoma [3]. Craniospinal irradiation after surgery has been suggested to improve long-term outcome in patients with medulloblastoma [1, 4, 5]. However, the development of radioresistance hampers therapeutic efficacy. Accumulating evidence suggests that cancer stem cells (CSCs) contribute to tumor radioresistance [6]. For instance, Yan et al. reported that AhR activation enhances cancer stem-like properties and radioresistance [7]. Shi et al.reported that pharmacological inhibition of bone marrow and X-linked (BMX) disrupts glioma stem cells and reduces radioresistance [8]. CD133 is a widely used CSC marker [9, 10]. Garg et al.reported that CD133^+^ CSCs contribute to medulloblastoma recurrence through the signal transducer and activator of transcription 3 (STAT3) signaling axis [10]. Identification of novel cancer stemness regulators is of significance in improving radiotherapy for medulloblastoma.

Sirtuin 6 (SIRT6) belongs to the sirtuin family of protein deacetylases and participates in various biological processes, including growth, differentiation, and inflammation [11–14]. SIRT6 is involved in base excision repair and thus contributes to genomic stability [12]. In hepatocellular carcinoma (HCC), SIRT6 can potentiate apoptosis resistance by repressing the transcription of the pro-apoptotic gene Bax [15]. Similarly, SIRT6 confers resistance to DNA damage in multiple myeloma cells through downregulation of mitogen-activated protein kinase (MAPK) pathway genes [16]. Depletion of SIRT6 blocks DNA repair responses and enhances the sensitivity of acute myeloid leukemia cells to DNA-damaging agents [17]. Therefore, SIRT6 plays an important role in tumor progression.

Long non-coding RNAs (lncRNAs) are a family of regulatory RNA molecules of > 200 nucleotides in length [18]. Although they lack protein-coding potential, lncRNAs can interact with proteins or other RNA molecules to modulate gene expression and activity [19, 20]. For instance, the lncRNA ARHGAP5-AS1 enhances chemoresistance in gastric cancer cells through stabilization of ARHGAP5 mRNA [19]. Chen et al. reported that the lncRNA IHS activates the ERK and AKT signaling pathways to stimulate the proliferation and metastasis in HCC [20]. LncRNA RBM5-AS1 has been suggested as a critical modulator of colon cancer stemness [21]. Biochemically, RBM5-AS1 is localized in the nucleus of colon cancer cells and can directly interact with β-catenin to enhance the transcription of specific β-catenin targets [21]. The upregulation of β-catenin by RBM5-AS1 has also been noted in bone cells [22]. Li et al. reported that RBM5-AS1 can promote the proliferation and invasion of oral squamous cell carcinoma cells via the miR-1285-3p/YAP1 axis [23]. However, the biological role of RBM5-AS1 in medulloblastoma remains unclear.

In the present study, we profiled CSC-related lncRNAs including RBM5-AS1 between radioresistant and parental medulloblastoma cells. The function of RBM5-AS1 in the growth, stemness, and radiosensitivity of medulloblastoma cells was clarified. The RBM5-AS1-interacting partner was also investigated.

## Results

### RBM5-AS1 depletion sensitizes medulloblastoma cells to radiation treatment

**Fig. 1:**
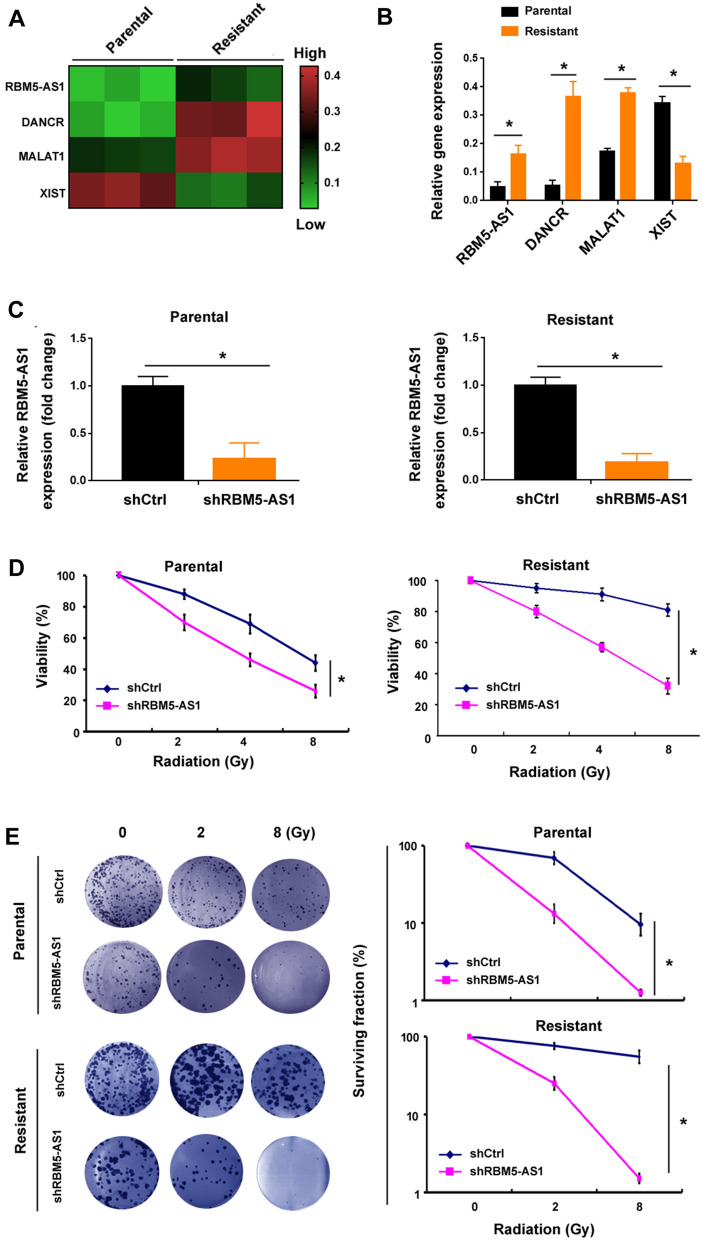
RBM5-AS1 depletion sensitizes medulloblastoma cells to radiation treatment. **A** Quantitative real-time PCR arrays were performed to identify dysregulated lncRNAs between radioresistant and parental DAOY cells. The heatmap shows the lncRNAs that significantly differ between the 2 groups. **B** Bar graphs show the level of RBM5-AS1, DANCR, MALAT1, and XIST between radioresistant and parental DAOY cells. **C** Measurement of RBM5-AS1 levels in radioresistant and parental DAOY cells transfected with control shRNA (shCtrl) or shRBM5-AS1. **D** Radioresistant and parental DAOY cells in serum-free medium were exposed to different doses of X-rays, and cell viability was measured after 3 days. **E** Clonal formation assays showed that RBM5-AS1 knockdown increased the sensitivity of radioresistant and parental DAOY cells to radiation. *Left* panels: representative photographs of dishes with colonies. *P < 0.05

To identify lncRNAs involved in radioresistance of medulloblastoma cells, we profiled 84 CSC-related lncRNAs in radioresistant and parental DAOY cells using quantitative real-time PCR arrays. CSC-related lncRNAs were chosen given the causal relationship between cancer stemness and radioresistance [7, 8]. Among the lncRNAs tested (Additional file 1: Table S1), 4 lncRNAs showed significant expression changes: i.e., XIST with 2.6-fold downregulation and RBM5-AS1, DANCR, and MALAT1 with 3.5-, 6.9-, and 2.2-fold upregulation, respectively (Fig. 1A, B). To ascertain the role of these deregulated lncRNAs in the radiosensitivity of medulloblastoma cells, we knocked down RBM5-AS1, DANCR, and MALAT1 and overexpressed XIST in DAOY cells. Silencing of RBM5-AS1 increased the radiosensitivity of radioresistant and parental DAOY cells (Fig. 1C, D). However, depletion of DANCR or MALAT1 or overexpression of XIST did not affect the radiosensitivity of radioresistant DAOY cells (Additional file 1: Figure S1). Clonogenic survival assay further demonstrated that knockdown of RBM5-AS1 significantly diminished surviving fraction after radiation in radioresistant and parental DAOY cells (Fig. 1E). These results suggest that RBM5-AS1 plays a critical role in radiation resistance of medulloblastoma cells.

**Fig. 2:**
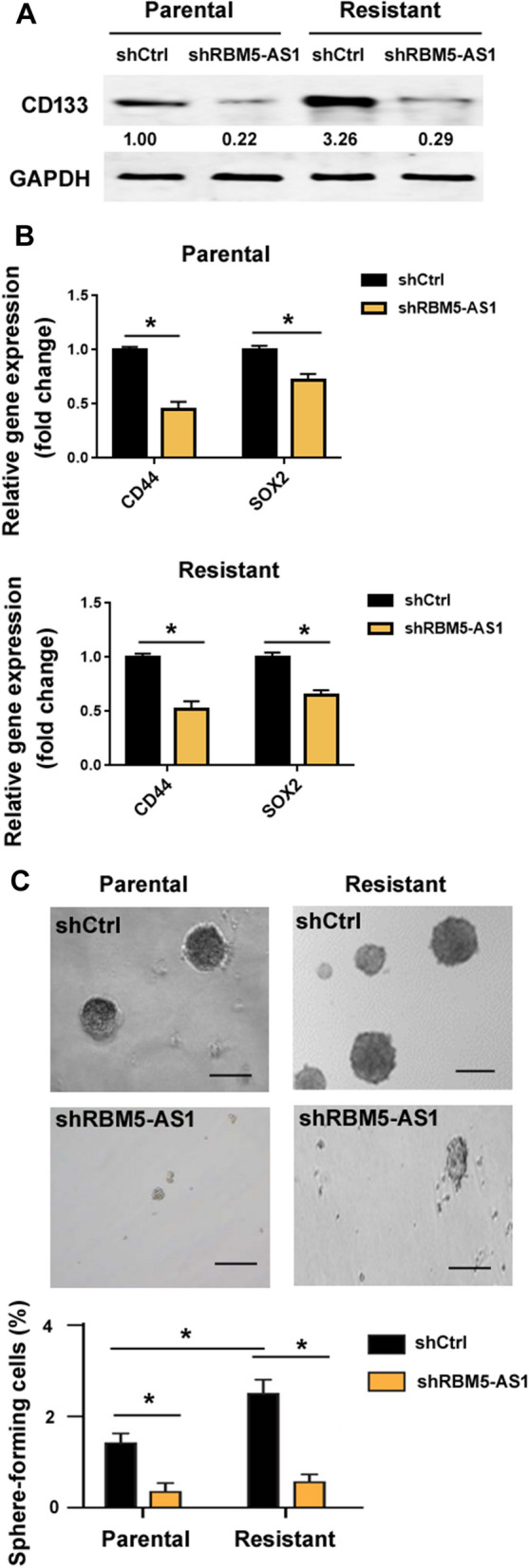
RBM5-AS1 contributes to medulloblastoma stemness. **A** Western blot analysis of the stem cell marker CD133 in radioresistant and parental DAOY cells transfected with control shRNA (shCtrl) or shRBM5-AS1. **B** Quantitative real-time PCR analysis of CD44 and SOX2 in radioresistant and parental DAOY cells transfected with indicated constructs. **C** Radioresistant and parental DAOY cells transfected with shCtrl or shRBM5-AS1 were cultured in the suspension condition to allow the formation of tumorspheres. *Top* panels: representative photographs of tumorspheres. Scale bar = 40 μm. *P < 0.05

Next, we validated the role of RBM5-AS1 in the maintenance of medulloblastoma stemness. The stem cell marker CD133 has been used to identify medulloblastoma stem cells [9, 10]. Analysis of CD133 expression showed that radioresistant DAOY cells expressed a higher level of CD133 than parental cells (Fig. 2A). Moreover, knockdown of RBM5-AS1 led to a reduction in the level of CD133 in radioresistant DAOY cells. We also investigated the effect of RBM5-AS1 on the expression of other stemness markers CD44 and SOX2. As shown in Fig. 2B, RBM5-AS1 depletion decreased the expression of both CD44 and SOX2 in parental and radioresistant DAOY cells. Sphere-forming assay was then used to determine stem cell self-renewal. We found that knockdown of RBM5-AS1 impaired the formation of spheres by radioresistant DAOY cells (Fig. 2C). These findings confirm the regulation of medulloblastoma stemness by RBM5-AS1.

### RBM5-AS1 knockdown induces apoptosis and DNA damage response

**Fig. 3:**
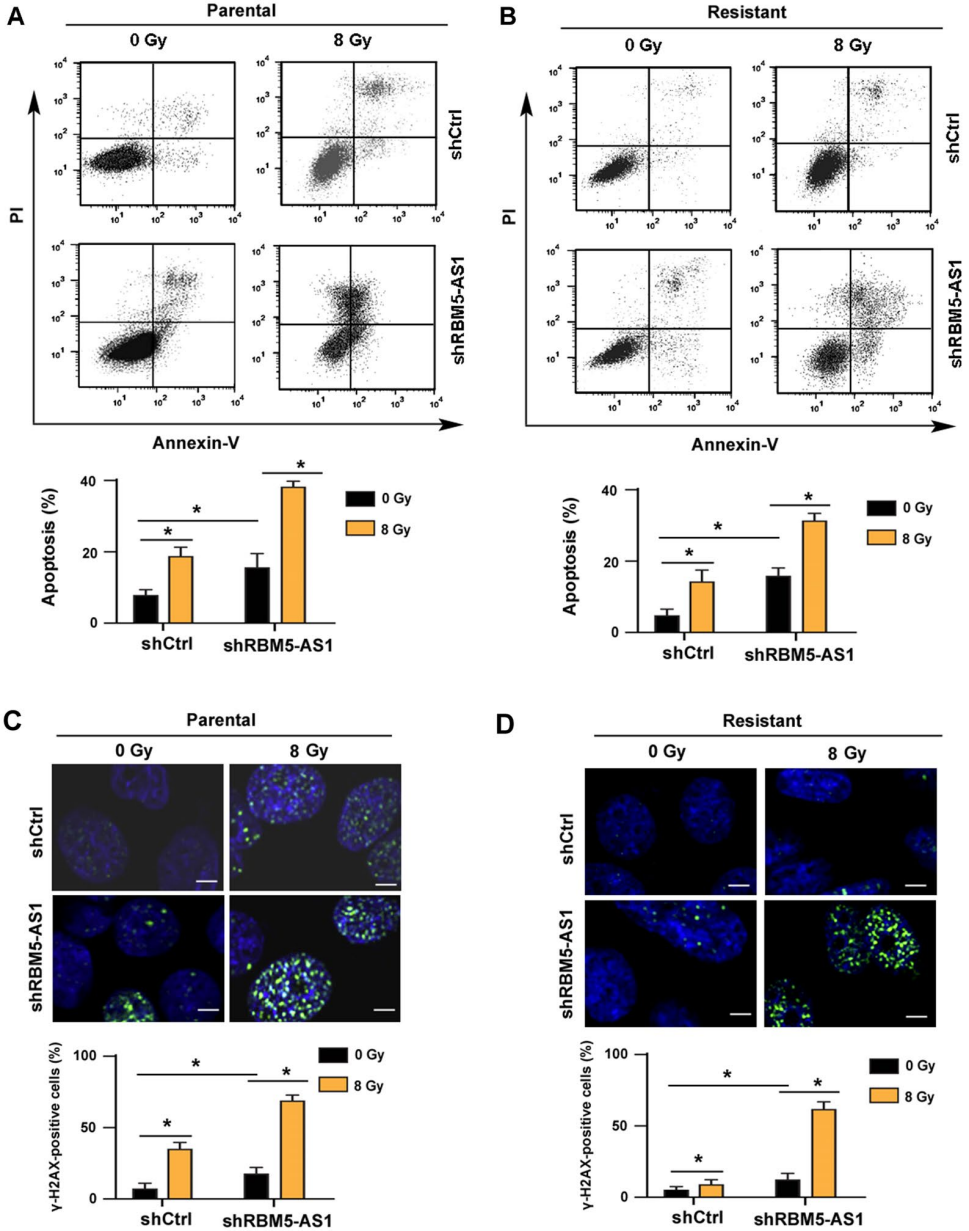
RBM5-AS1 knockdown induces apoptosis and DNA damage response. **A**, **B** Cells transfected with shCtrl or shRBM5-AS1 were radiated with X-rays at 8 Gy. Apoptosis was measured using Annexin-V/PI staining. **C**, **D** DNA damage response was evaluated by immunostaining for γ-H2AX. Scale bar = 20 μm. *P < 0.05

Next, we investigated the effects of knockdown of RBM5-AS1 on apoptosis and DNA damage response in medulloblastoma cells. We found that radiation exposure led to a significant apoptosis in parental DAOY cells, which was enhanced by knockdown of RBM5-AS1 (Fig. 3A). Similarly, silencing of RBM5-AS1 significantly enhanced radiation-induced apoptosis in radioresistant DAOY cells (Fig. 3B). DNA damage response was evaluated by examining the expression of γ-H2AX, a biomarker of double strand breaks [24]. Of note, RBM5-AS1 silencing reinforced DNA damage response after radiation (Fig. 3C, D). Taken together, these findings suggest that knockdown of RBM5-AS1 augments radiation-induced apoptosis and DNA damage responses.

### Depletion of RBM5-AS1 suppresses tumor growth and increases radiosensitivity in vivo

**Fig. 4:**
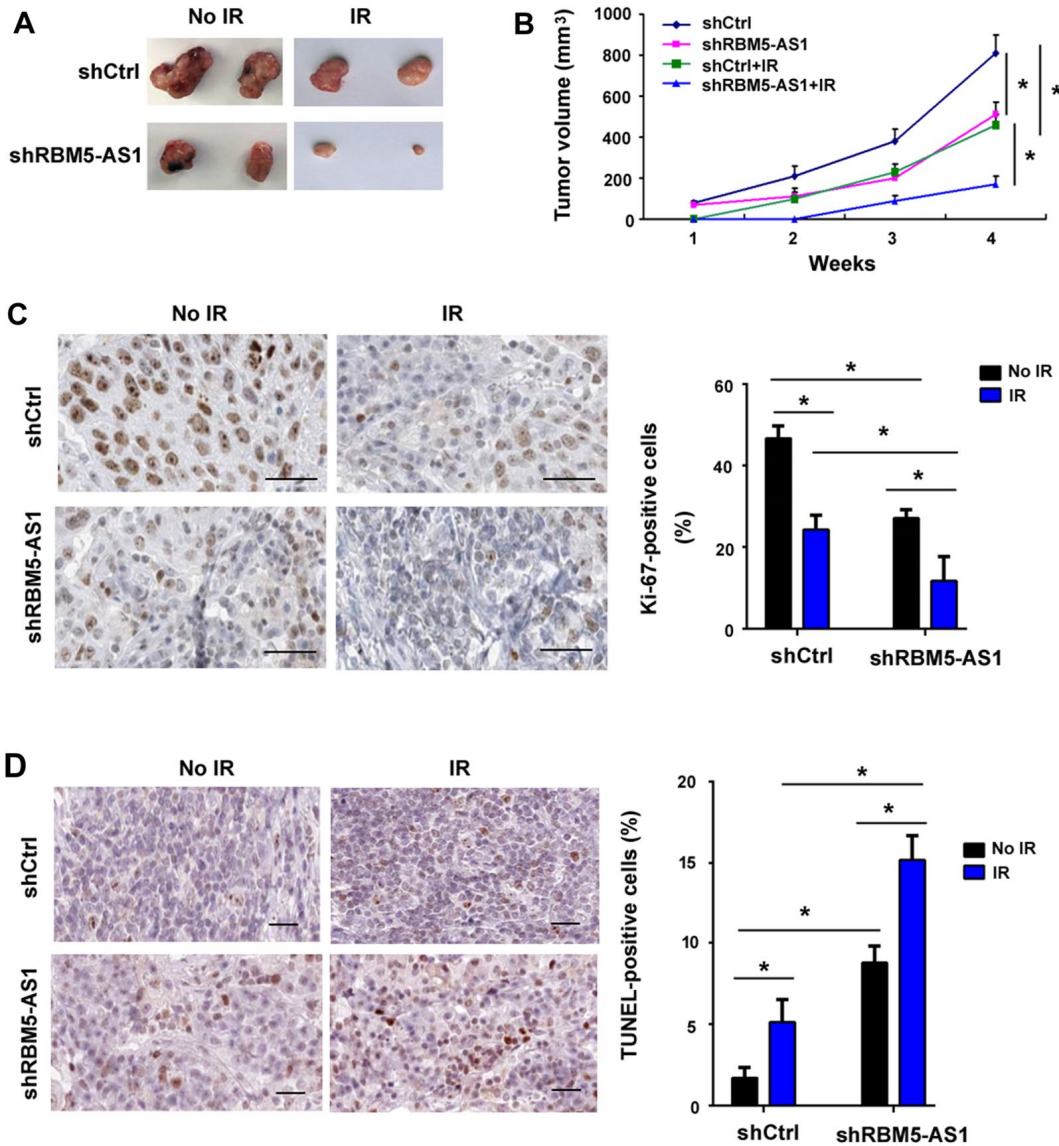
Depletion of RBM5-AS1 suppresses tumor growth and increases radiosensitivity in vivo. **A**, **B** DAOY xenograft tumors were exposed to irradiation (IR), and tumor growth was determined. **A** Macroscopic view of xenograft tumors. **C** Immunohistochemical staining for Ki-67 in xenograft tumor sections. Scale bar = 100 μm. (D) TUNEL staining in xenograft tumor sections. Scale bar = 100 μm. *P < 0.05

Next, we validated the effects of depletion of RBM5-AS1 on tumor growth and radiosensitivity in athymic nude mice. As shown in Fig. 4A, depletion of RBM5-AS1 reduced the growth of DAOY xenograft tumors. Furthermore, RBM5-AS1-depleted DAOY xenograft tumors showed more sensitive to radiation exposure than control tumors (Fig. 4A, B). Immunohistochemical staining indicated a reduction of Ki-67-positive proliferative cells (Fig. 4C) and increase of terminal dUTP nick-end labeling (TUNEL)-positive apoptotic cells (Fig. 4D) in the RBM5-AS1-depleted tumors after radiation treatment. Taken together, RBM5-AS1 knockdown restrains tumor growth and enhances radiosensitivity in medulloblastoma.

### Ectopic expression of RBM5-AS1 confers radioresistance to medulloblastoma cells

**Fig. 5:**
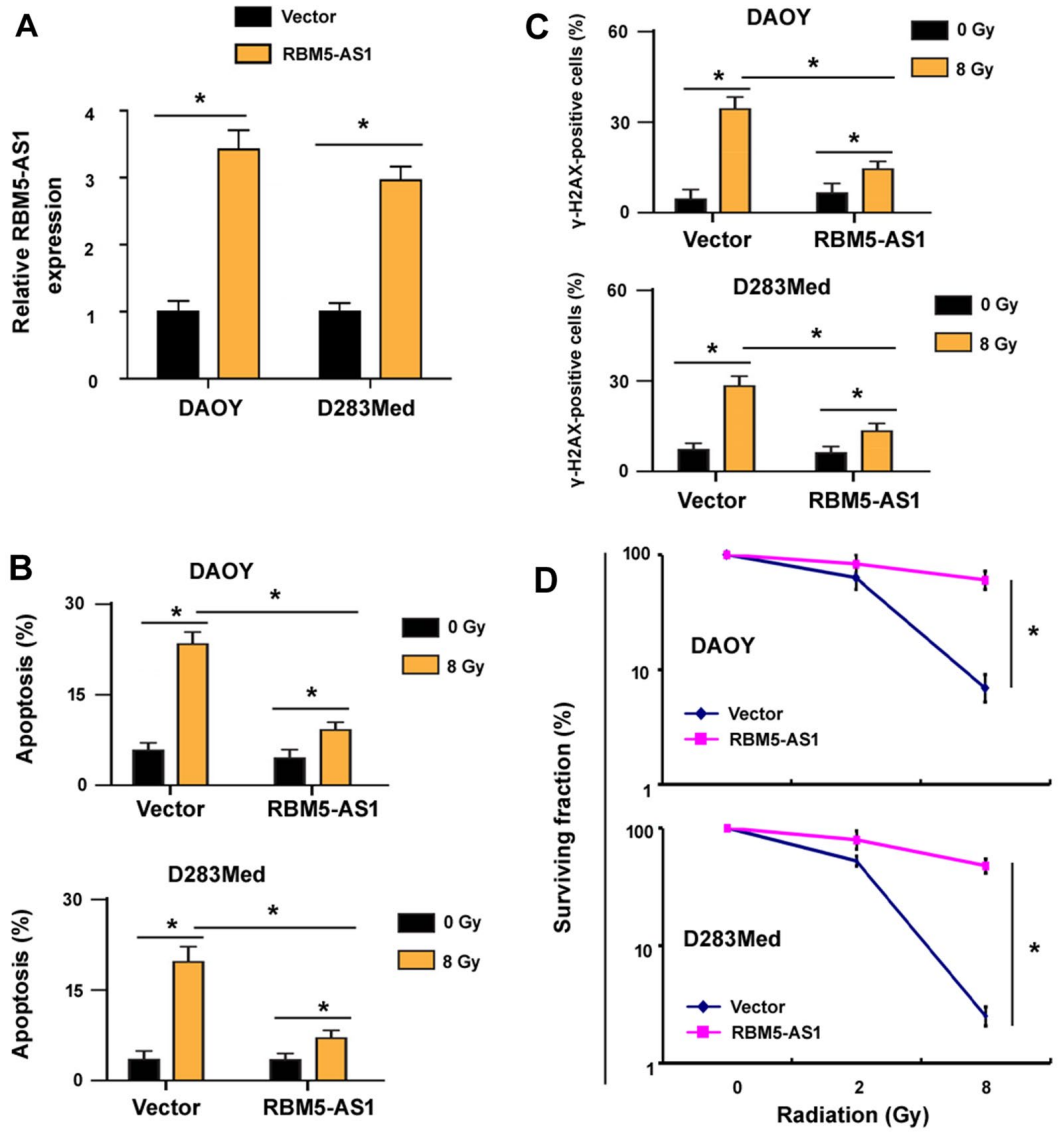
Ectopic expression of RBM5-AS1 confers radioresistance to medulloblastoma cells. **A** Quantitative real-time PCR analysis of RBM5-AS1 levels in both DAOY and D283Med cells transfected with empty vector or RBM5-AS1-expressing plasmid. **B** Detection of apoptosis in DAOY and D283Med cells transfected with empty vector or RBM5-AS1-expressing plasmid after irradation. **C** Assessment of DNA damage response in the cells treated as in **B**. **D** Clonogenic survival assay showing that RBM5-AS1-overexpressing cells were more resistant to radiation than control cells. *P < 0.05

Next, we asked whether overexpression of RBM5-AS1 could induce radioresistance in medulloblastoma cells. We observed that enforced expression of RBM5-AS1 (Fig. 5A) attenuated radiation-induced apoptosis in DAOY cells (Fig. 5B), compared to empty vector-transfected medulloblastoma cells. Similar findings were noted in D283Med medulloblastoma cells (Fig. 5A, B). Moreover, overexpression of RBM5-AS1 protected medulloblastoma cells from radiation-induced DNA damage (Fig. 5C). In addition, clonogenic survival assay demonstrated that RBM5-AS1-overexpressing cells were more resistant to radiation than control cells (Fig. 5D). These data collectively indicate that RBM5-AS1 overexpression induces radioresistance in medulloblastoma cells.

### RBM5-AS1 associates with SIRT6 protein in medulloblastoma cells

**Fig. 6:**
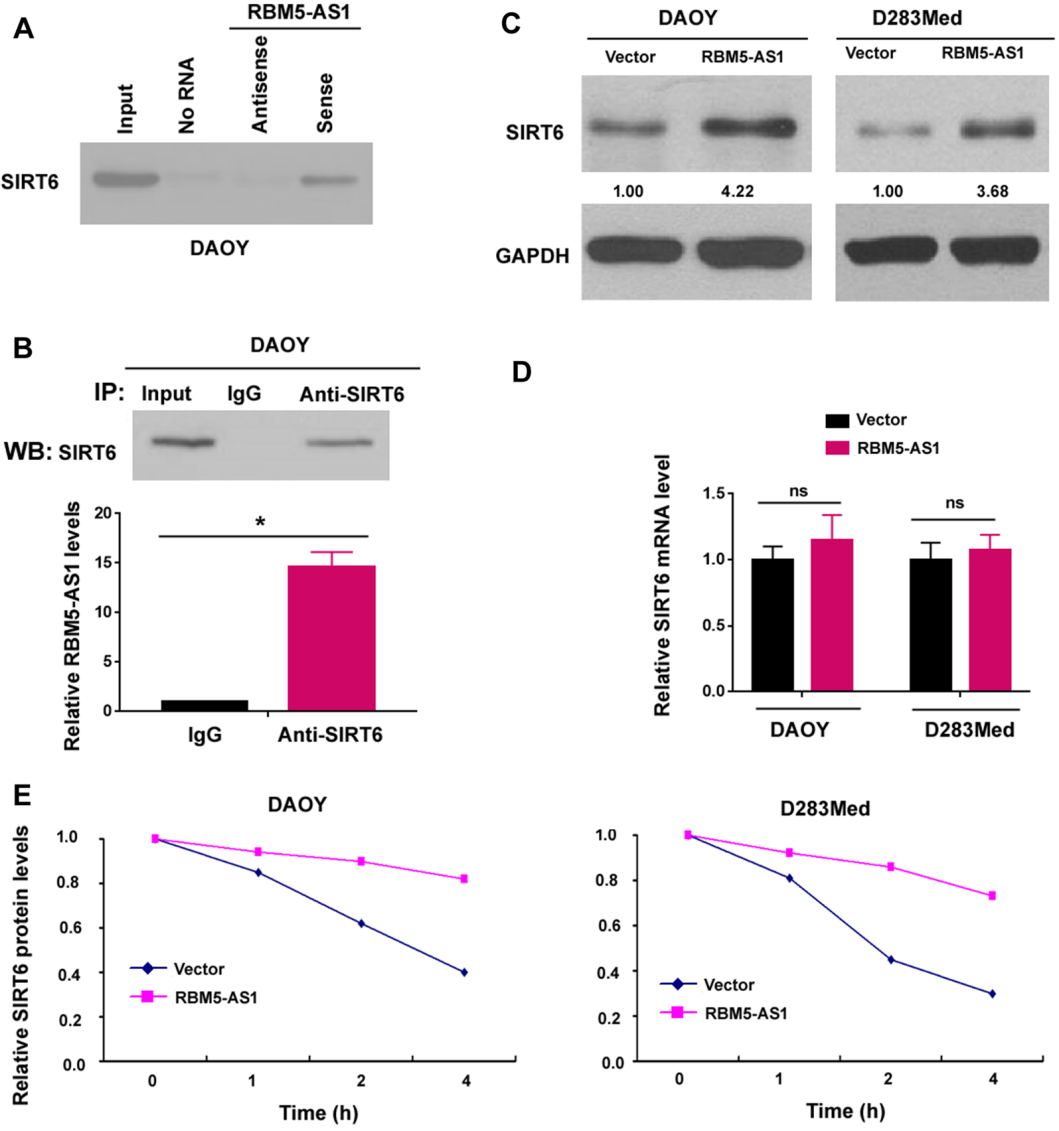
RBM5-AS1 associates with SIRT6 protein in medulloblastoma cells. **A** Western blot analysis of SIRT6 protein in the RNA–protein complex that was pulled down by RBM5-AS1 probes. **B** RIP assay performed with anti-SIRT6 antibody. The levels of RBM5-AS1 in immunoprecipitated samples were detected. *P < 0.05. **C** Western blot analysis showed that overexpression of RBM5-AS1 increased the protein level of SIRT6 in both DAOY and D283Med cells transfected with empty vector or RBM5-AS1-expressing plasmid. **D** Quantitative real-time PCR analysis of SIRT6 mRNA levels in the cells treated as in **C**. *ns* no significance. **E** Cycloheximide (CHX) was used to block protein synthesis, and SIRT6 protein stability was assessed

A previous study has shown that RBM5-AS1 directly interacts with β-catenin and promotes β-catenin activation in colon cancer cells [21]. However, we did not observe the enhanced activation of β-catenin signaling by RBM5-AS1 in medulloblastoma cells (Additional file 1: Figure S2). To determine the mechanism by which RBM5-AS1 regulates the radioresistant phenotype of medulloblastoma cells, we performed RBM5-AS1 pulldown assays. RBM5-AS1 interacting proteins were identified by mass spectrometry. We validated the presence of SIRT6 in the RBM5-AS1 pulldown complex by Western blot analysis (Fig. 6A). In this study, SIRT6 was selected for further validation because of its importance in DNA damage response [15–17]. We performed RNA immunoprecipitation (RIP) assay using anti-SIRT6 antibody. Compared to control IgG immunoprecipitated sample, the SIRT6 antibody-bound complex contained a significantly greater level of RBM5-AS1 (Fig. 6B).

Given the interaction between RBM5-AS1 and SIRT6, we asked whether RBM5-AS1 could modulate the expression of SIRT6 in medulloblastoma cells. Notably, we found that overexpression of RBM5-AS1 led to an increase in the protein level of SIRT6 in both DAOY and D283Med cells (Fig. 6C). However, RBM5-AS1 overexpression did not affect the mRNA level of SIRT6 (Fig. 6D), suggesting that RBM5-AS1 modulates the expression of SIRT6 at post-transcriptional level. Next we asked whether RBM5-AS1-mediated upregulation of SIRT6 expression was a result of increased protein stability. To address this, we used cycloheximide (CHX) to block protein synthesis. As expected, overexpression of RBM5-AS1 increased the stability of SIRT6 protein (Fig. 6E). Taken together, RBM5-AS1 associates with and stabilizes SIRT6 protein in medulloblastoma cells.

### SIRT6 mediates the oncogenic activity of RBM5-AS1 in medulloblastoma

**Fig. 7:**
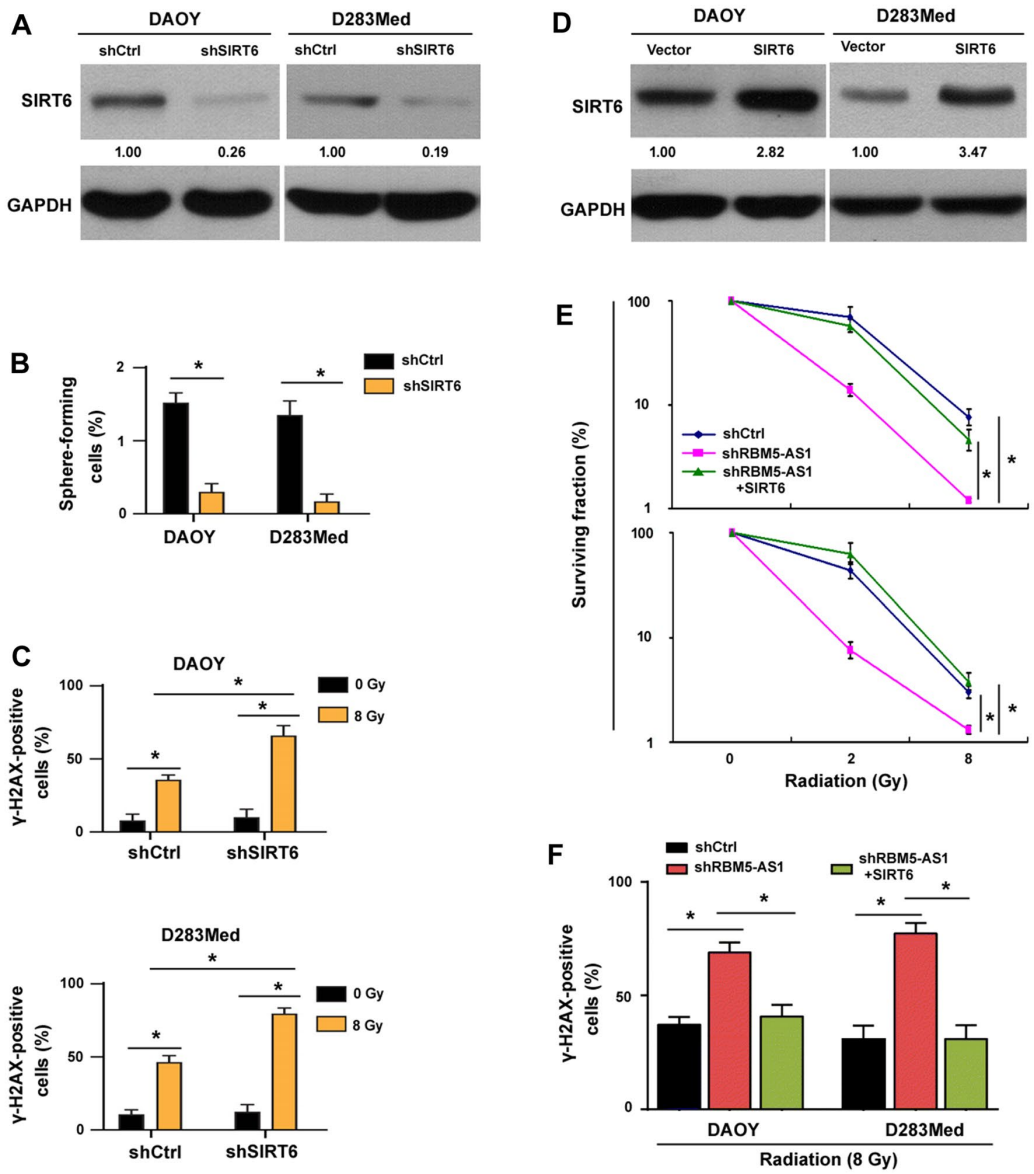
SIRT6 mediates the oncogenic activity of RBM5-AS1 in medulloblastoma. **A** Western blot analysis showed depletion of SIRT6 in medulloblastoma cells. **B** Effect of SIRT6 knockdown on self-renewal capacity of cancer stem cells. **C** Depletion of SIRT6 enhanced radiation-induced DNA damage. **D** Western blot analysis confirmed overexpression of SIRT6 in medulloblastoma cells. **E** Overexpression of SIRT6 rescued RBM5-AS1 depletion-induced radiosensitization. **F** Overexpression of SIRT6 attenuated RBM5-AS1 depletion-induced DNA damage response. *P < 0.05

To investigate the importance of SIRT6 in RBM5-AS1-mediated aggressive phenotype, we knocked down SIRT6 in medulloblastoma cells (Fig. 7A). Similar to the phenotype of RBM5-AS1-depleted cells, depletion of SIRT6 reduced the stem-like properties (Fig. 7B) and enhanced radiation-induced DNA damage (Fig. 7C) in medulloblastoma cells. In addition, overexpression of SIRT6 (Fig. 7D) reversed RBM5-AS1 depletion-induced radiosensitization (Fig. 7E) and DNA damage (Fig. 7F). Thus, SIRT6 is functionally relevant to RBM5-AS1 in medulloblastoma.

## Discussion

Several lncRNAs have been reported to contribute to medulloblastoma growth and survival [25, 26]. For instance, silencing of the lncRNA TP73-AS1 induces apoptosis and inhibits cell proliferation and migration in medulloblastoma cells [27, 28]. In this study, we identify a novel medulloblastoma driver lncRNA. We show that RBM5-AS1 is upregulated in radioresistant DAOY cells (Fig. 1A). Depletion of RBM5-AS1 overcomes the radioresistance of medulloblastoma cells, as evidenced by reduced cell viability and clonogenic survival after exposure to radiation (Fig. 1C, D). Conversely, ectopic expression of RBM5-AS1 enhances clonogenic survival of medulloblastoma cells in response to radiation (Fig. 5). Our findings provide first evidence for the role of RBM5-AS1 in inducing radioresistance in medulloblastoma.

It has been suggested that CSCs play a critical role in the development of radioresistance [7, 8]. Chen et al. reported that CD133^+^ CSCs are enriched upon radiation and confer radioresistance in non-small cell lung cancer [29]. JARID1B silencing-mediated suppression of stemness results in increased radiation sensitivity in oral carcinoma [30]. It has been previously demonstrated that RBM5-AS1 is involved in the enrichment of colon cancer CSCs [21]. Consistently, RBM5-AS1 also promotes the self-renewal of medulloblastom CSCs (Fig. 2B). Moreover, knockdown of RBM5-AS1 reduces the expression of the CSC marker CD133 (Fig. 2A). These findings suggest that RBM5-AS1-mediated radioresistance may be causally linked to enhanced cancer stemness. It has been documented that the population of medulloblastoma CD133^+^ cell is enlarged upon hypoxic stimulation, and CD133^+^ cells exhibit more radioresistant than CD133^−^ cells [31]. Therefore, it is interesting to determine the role of RBM5-AS1 in medulloblastoma radioresistance under hypoxic conditions.

Previous studies have indicated RBM5-AS1 as an oncogene in colon cancer and oral squamous cell carcinoma [21, 23]. Li et al. reported that RBM5-AS1 has the ability to promote oral squamous cell carcinoma cell proliferation and invasion [23]. In this study, we show that RBM5-AS1 knockdown enhances radiation-induced apoptosis in medulloblastoma cells (Fig. 3A, B). However, radiation caused comparable apoptosis in radioresistant and parental DAOY cells transfected with control shRNA, which may be due to short observation time, i.e. 48 h after X-ray exposure. Indeed, we noted that the parental group had significantly profound apoptosis than the radioresistant group 72 h after radiation (data not shown). We further demonstrate that overexpression of RBM5-AS1 protects medulloblastoma cells from radiation-induced apoptosis (Fig. 5). Consistently, ectopic expression of RBM5-AS1 attenuates radiation-induced DNA damage in medulloblastoma cells (Fig. 5C). These results confirm the prosurvival capacity of RBM5-AS1 in medulloblastoma cells. In agreement with our data, several lncRNAs such as Meg3 and PVT1 have been found to regulate DNA damage repair and apoptosis responses [32, 33]. We also validated the role of RBM5-AS1 in the xenograft mouse model. Of note, depletion of RBM5-AS1 slows the growth of DAOY xenograft tumors (Fig. 4). Moreover, RBM5-AS1 silencing sensitizes DAOY xenograft tumors to radiation, which is associated with increased apoptosis. Therefore, RBM5-AS1 may represent a promising target to overcome radioresistance in medulloblastoma.

lncRNAs exert their biological activities via multiple mechanisms, such as sponging of microRNAs [23], epigenetic silencing of gene expression [34], and posttranslational modification of proteins [35]. Di Cecilia et al. revealed the interaction between RBM5-AS1 and β-catenin protein in colon cancer cells [21]. However, such interaction was not detected in medulloblastoma cells, suggesting another mechanism involved in the oncogenic activity of RBM5-AS1 in medulloblastoma. Specially, we discover that RBM5-AS1 interacts with SIRT6 protein in medulloblastoma cells (Fig. 6). RBM5-AS1 overexpression increases the protein levels, which is at least, in part, a result of increased stabilization of SIRT6 protein. The lncRNA HULC has been shown to stabilize SIRT1 protein through induction of ubiquitin-specific peptidase (USP) 22 and thus inhibition of ubiquitin-mediated SIRT1 degradation [35]. USP10 has exhibited the ability to prevent SIRT6 protein from ubiquitination and degradation [36, 37]. These studies suggest the possibility that the association with RBM5-AS1 may reinforce USP10-dependent suppression of SIRT6 degradation. However, the detailed mechanism for RBM5-AS1-mediated stabilization of SIRT6 remains to be uncovered in future work.

SIRT6 has been extensively reported to modulate tumor progression [13, 14, 38]. For example, SIRT6 contributes to the invasiveness and metastasis in lung cancer and ovarian cancer [14, 39]. Cagnetta et al. has indicated that SIRT6 mediates DNA repair in leukemia [17]. Similarly, Lee et al. reported that SIRT6 prevents DNA damage and cellular senescence in HCC cells [40]. Our data confirm the role of SIRT6 in the regulation of DNA damage response of medulloblastoma cells (Fig. 7). We show that knockdown of SIRT6 enhances radiation-induced DNA damage, which is similar to the finding in RBM5-AS1-depleted cells. Moreover, enforced expression of SIRT6 reverses RBM5-AS1 depletion-induced radiosensitization and DNA damage response. However, the key pathways involved in SIRT6 oncogenic activity remain to be clarified. There is evidence that SIRT6 safeguards human mesenchymal stem cells from oxidative stress [41]. Consistently, our data indicate that SIRT6 knockdown impairs the stemness of medulloblastoma cells. However, a previous study reported that SIRT6 overexpression inhibits cancer stem-like capacity in breast cancer with PI3K activation [42]. Therefore, SIRT6 likely plays distinct roles in different origins of stem cells. Nevertheless, SIRT6 mediates the oncogenic activity of RBM5-AS1 in medulloblastoma through promotion of stem-like capacity and attenuation of DNA damage. These findings warrant investigation of the therapeutic potential of targeting both RBM5-AS1 and SIRT6 in medulloblastoma.

Although in this study we indicate that RBM5-AS1 regulates growth and radioresistance of medulloblastoma cells, the clinical significance of RBM5-AS1 has not been addressed yet. Future work is needed to explore the expression, tissue distribution, and clinical relevance of RBM5-AS1 in medulloblastoma. Moreover, direct evidence is required to confirm the causal link between RBM5-AS1-induced stemness and radioresistance of medulloblastoma cells. In addition, the findings in radioresistant DAOY cells will be validated in other radioresistant medulloblastoma cell lines.

In conclusion, RBM5-AS1 is a radiation responsive lncRNA that contributes to increased stemness and radioresistance in medulloblastoma. The oncogenic activity of RBM5-AS1 is ascribed to stabilization of SIRT6. Thus, targeting RBM5-AS1 may offer a potential strategy for improving radiotherapy in medulloblastoma.

## Materials and methods

### Cell culture

Medulloblastoma cell lines DAOY and D283Med were obtained from the American Type Culture Collection (Manassas, VA, USA) and cultured in Dulbecco’s modified Eagle’s medium (DMEM; Sigma-Aldrich, St. Louis, MO, USA) containing 10% fetal bovine serum (FBS; HyClone, Logan, UT, USA) at 37 °C in 5% CO_2_. No mycoplasma contamination was found.

### Establishment of radioresistant cell lines

Radioresistant DAOY-IR cells were developed as described previously [43]. Briefly, DAOY cells were repeatedly exposed to 4 Gy of X-rays. After treatment with a cumulative dose of 80 Gy, the surviving cell clones, namely DAOY-IR, were recovered and expanded.

### Quantitative real-time PCR analysis

Total RNA was extracted from cells using TRIzol reagent (Invitrogen, Grand Island, NY, USA) following the manufacturer's instructions. Reverse transcription was performed using the SuperScript III Reverse Transcriptase kit (Invitrogen). Quantitative PCR was then done using the following PCR primers: *RBM5-AS1* forward: 5′-GCTTCAACACTGCGTGACAA-3′, reverse: 5′-CGTGGAATCAAATGGAGTGG-3′; *SIRT6* forward: 5′-CGTGGATGAGGTGATGTG-3′, reverse: 5′-GGCTTATAGGAACCATTGAGA-3′; *CD44* forward: 5′-GCCCAATGCCTTTGATGGACC-3′, reverse: 5′-GCAGGGATTCTGTCTGTGCTG-3′; *SOX2* forward: 5′-GCCTGGGCGCCGAGTGGA-3′, reverse: 5′-GGGCGAGCCGTTCATGTAGGTCTG-3′; *B-Actin* forward: 5′-GGTGGCTTTTAGGATGGCAAG-3′, reverse: 5′-ACTGGAACGGTGAAGGTGACAG-3′. We also performed quantitative real-time PCR arrays to profile 84 CSC-related lncRNAs in radioresistant and parental DAOY cells. The candidate lncRNAs are listed in Additional file 1: Table S1. The relative gene expression was calculated by the 2^−ΔΔCt^ method [44].

### Plasmids and cell transfection

RBM5-AS1- and SIRT6-targeting short hairpin RNAs (shRNAs) were synthesized by Beijing Hanyu Biomed (Beijing, China) and cloned to pLKO.1 vector (Sigma-Aldrich). The targeting sequence for RBM5-AS1 and SIRT6 was 5′-GAGUCACAUUCCUUAGCCAUG-3′ and 5′-GACAAACUGGCAGAGCUCCAC-3′, respectively. The RBM5-AS1- and SIRT6-expressing plasmids were constructed by Beijing Hanyu Biomed. All plasmids were verified by DNA sequencing.

Cell transfection was performed using the Lipofectamine LTX Plus (Invitrogen) as per the manufacturer’s protocol. Twenty-four hours after transfection, transfected cells were subjected to gene expression analysis. For selection of stable cell lines, transfected cells were selected in the medium containing 1 μg/mL puromycin (Sigma-Aldrich).

### In vitro sphere-forming assay

Cells were seeded onto ultra-low attachment 24-well plates (Corning, Lowell, MA, USA) at a density of 1000 cells per well. They were cultured in serum-free DMEM/F12 medium supplemented with basic fibroblast growth factor and epidermal growth factor (20 ng/mL each; Invitrogen) for 2 weeks. Culture media were replenished every 3 days. The number of spheres in each well was determined.

### Cell viability assay

Cells in serum-free medium were exposed to different doses of X-rays and cultured for 3 days. Cell viability was measured using the 3-(4,5-dimethythiazol-2-yl)-2,5-diphenyl tetrazolium bromide (MTT) method. Cells were incubated with 0.5 mg/mL MTT (Sigma-Aldrich) at 37 °C for 4 h. After addition of dimethyl sulfoxide, absorbance was measured at 570 nm with a microplate spectrophotometer.

### Clonogenic assay

Cells were plated onto 6-well plates (4000 cells/well) overnight and then radiated with 2 or 8 Gy. Media were changed every 3 days until colonies were formed. Fifteen days later, cells were fixed with 4% of buffered formalin for 15 min and stained with 0.25% crystal violet for 20 min. The colonies were counted for each well. The surviving fraction was calculated as a ratio of the number of colonies divided by the total number of cells seeded.

### Flow cytometry analysis of apoptosis

Apoptotic response was evaluated 48 h after exposure to 8 Gy of X-rays. In brief, cells were washed and resuspended in the Annexin Binding Buffer. The cell suspension was added with Annexin V conjugated with fluorescein isothiocyanate (FITC; Sigma-Aldrich) and incubated for 15 min at 4 °C. Afterwards, the cells were stained with propidium iodide (PI; Sigma-Aldrich). Apoptotic cells were immediately analyzed using the FACScantoII cytometer with the FlowJo 10.2 software (BD Bioscience, San Jose, CA, USA).

### Immunofluorescent staining

DNA damage was assessed by analyzing γ-H2AX foci formation [45]. Briefly, cells were radiated with a dose of 8 Gy, fixed with 4% paraformaldehyde, and permeabilized with 0.2% TritonX-100. After blocking, the cells were incubated with anti-phospho-γ-H2AX (ab26350, Abcam, Cambridge, MA, USA; 1:50 dilution). An Alexa-Fluor 488 conjugated goat anti-rabbit IgG was used as the secondary antibody. Nuclei were counter-stained with 4′,6-diamidino-2-phenylindole (DAPI; Sigma-Aldrich).

### Animal studies

Five-week-old male BALB/c nude mice (nu/nu) were maintained in a pathogen-free environment and allowed free access to water and food. These mice were randomly assigned to 4 groups (5 mice each group) and received subcutaneous inoculation of RBM5-AS1-depleted or control DAOY cells (2 × 10^6^ cells/mouse) with or without radiation exposure. For radiation treatment, mice were given a single dose of 10 Gy on day 3 after cell injection. Tumor volume was calculated weekly for 4 weeks. After the last measurement, mice were sacrificed and tumors were removed. Tumor sections were deparaffinized with xylene and rehydrated by alcohol gradient. Endogenous peroxidase activity was blocked using a 0.3% hydrogen peroxide solution. The sections were subjected to immunostaining with anti-Ki-67 antibody (ab15580, Abcam; 1:100 dilution). For apoptosis analysis, TUNEL staining was performed on tumor sections with the TUNEL Staining kit (Beyotime, Haimen, China). The percentage of Ki-67- and TUNEL-positive cells was calculated in 5 random microscopic fields. All studies involving animals were approved by the Animal Care and Use Committee of Shanghai Jiaotong University School of Medicine (Shanghai, China; approval number: 2018-0269).

### RNA pulldown assay

Biotin-labeled sense or antisense RBM5-AS1 RNAs were generated by in vitro transcription using Biotin RNA Labeling Mix (Sigma-Aldrich) and T7 RNA polymerase (Promega, Madison, WI, USA). The labeled RNA was purified using the RNeasy Mini Kit (Qiagen, Valencia, CA, USA) and then incubated with precleared DAOY cell lysates at 4 °C overnight. The RNA–protein binding complexes were captured by streptavidin agarose beads (Invitrogen). The released proteins were detected by Western blot analysis or mass spectrometry.

### RIP assay

DAOY cells (2 × 10^6^ cells) were lysed in RIP immunoprecipitation buffer supplemented with RNase inhibitors (Invitrogen). The RIP lysates were centrifuged, and the supernatant was incubated at 4 °C overnight with magnetic beads conjugated with anti-SIRT6 antibody (ab191385, Abcam; 1:20 dilution) or negative control IgG (Abcam). The immunoprecipitate was treated with proteinase K at 55 °C for 30 min. The precipitated RNA samples were tested for RBM5-AS1 by quantitative PCR analysis.

### Western blot analysis

Cells were lysed using Radioimmunoprecipitation Assay Buffer (RIPA) supplemented with a protease inhibitor cocktail (Sigma-Aldrich). Protein concentrations were determined using the Bio-Rad Protein Assay Kit (Bio-Rad, Hercules, CA, USA). Protein samples were separated by 12% sodium dodecyl sulphate‐polyacrylamide gel electrophoresis and transferred onto nitrocellulose membranes. The membranes were incubated with primary antibodies recognizing CD133 (ab19898, Abcam; 1:1000 dilution), SIRT6 (1:500 dilution) or GAPDH (ab181602, Abcam; 1:5000 dilution) overnight at 4 °C, followed by a horseradish peroxidase-conjugated secondary antibody (Sigma-Aldrich). The protein bands were visualized using the ECL Plus Chemiluminescence Detection Kit (Thermo Fisher Scientific, Rockford, IL, USA).

### Statistics

Results are expressed as mean ± standard deviation and were analyzed by the Student’s *t* test or one-way ANOVA followed by the Bonferroni’s test. *P* < 0.05 was considered statistically significant.

## Supplementary Information

**Additional file 1.** Supplementary materials.
