# Supplementary material for: RBM5-AS1 promotes radioresistance in medulloblastoma through stabilization of SIRT6 protein
Source: Acta Neuropathol Commun. 2021 Jul 5;9:123. doi: 10.1186/s40478-021-01218-2 (PMC8256544; doi:10.1186/s40478-021-01218-2)
Supplement: Supplementary file 1 — Additional file 1. Supplementary materials. [file 40478_2021_1218_MOESM1_ESM.doc]

**Supplementary Table S1. List of the lncRNAs tested in this study.**

| **Array ID** | **Gene name** | **Accession number** | **Mean fold change**  **(relative to control)** |
| --- | --- | --- | --- |
| 1 | MIR31HG | NR_027054 | 1.8 |
| 2 | LNCRNA-ATB | NR_160525 | 1.6 |
| 3 | MNX1-AS1 | NR_038835 | -1.5 |
| 4 | CAHM | NR_037593 | -1.7 |
| 5 | GUARDIN | NR_132738 | 1.2 |
| 6 | RBM5-AS1 | NR_045388 | 3.5 |
| 7 | DBH-AS1 | NR_102735 | 1.0 |
| 8 | DLEU2 | NR_152566 | 1.7 |
| 9 | DLX6-AS1 | NR_015448 | 1.6 |
| 10 | EGFR-AS1 | NR_047551 | 1.8 |
| 11 | DANCR | NR_024031 | 6.9 |
| 12 | EPB41L4A-AS2 | NR_027706 | -1.2 |
| 13 | EWSAT1 | NR_026949 | 1.9 |
| 14 | LINC00115 | NR_024321 | -1.4 |
| 15 | FAM74A3 | NR_026801 | 1.3 |
| 16 | FAS-AS1 | NR_028371 | 1.1 |
| 17 | FENDRR | NR_033925 | 1.7 |
| 18 | FER1L4 | NR_119376 | 1.5 |
| 19 | FEZF1-AS1 | NR_036484 | 1.2 |
| 20 | CASC11 | NR_117101 | 1.1 |
| 21 | CCAT1 | NR_108049 | 1.0 |
| 22 | CCAT2 | NR_109834 | 1.0 |
| 23 | CASC9 | NR_103848 | 1.4 |
| 24 | CASC8 | NR_117100 | 1.0 |
| 25 | CASC2 | NR_026939 | 1.1 |
| 26 | LNCOC1 | NR_038925 | 1.2 |
| 27 | CCEPR | NR_131782 | 1.4 |
| 28 | HOTAIR | NR_047517 | 1.5 |
| 29 | CRNDE | NR_034105 | 1.7 |
| 30 | LINC0094 | NR_040245 | 1.8 |
| 31 | MALAT1 | NR_002819 | 2.2 |
| 32 | HOTTIP | NR_037843 | 1.6 |
| 33 | WSPAR | NR_131252 | 1.5 |
| 34 | H19 | NR_002196 | 1.8 |
| 35 | RMST | NR_024037 | 1.2 |
| 36 | CLMAT3 | NR_109873 | 1.1 |
| 37 | FGF14-AS2 | NR_036487 | 1.0 |
| 38 | FOXCUT | NR_125804 | 1.0 |
| 39 | LINC-ROR | NR_048536 | 1.4 |
| 40 | XIST | NR_001564 | -2.6 |
| 41 | FOXD2-AS1 | NR_026878 | 1.0 |
| 42 | HCG11 | NR_026790 | -1.3 |
| 43 | CTBP1-AS | NR_104331 | -1.3 |
| 44 | IATPR | NR_160030 | -1.2 |
| 45 | HEIH | NR_045680 | 1.0 |
| 46 | BAIAP2-AS1 | NR_026857 | 1.1 |
| 47 | UCA1 | NR_015379 | 1.5 |
| 48 | AGAP2-AS1 | NR_027032 | -1.4 |
| 49 | PVT1 | NR_003367 | 1.7 |
| 50 | LINC00617 | NR_132398 | 1.0 |
| 51 | PRDM16-DT | NR_015440 | 1.0 |
| 52 | LINC00963 | NR_038955 | 1.0 |
| 53 | BACE1-AS | NR_037803 | 1.1 |
| 54 | AFAP1-AS1 | NR_026892 | -1.8 |
| 55 | LINC00184 | NR_033927 | 1.4 |
| 56 | LINC00261 | NR_001558 | 1.1 |
| 57 | FAM30A | NR_026800 | 1.0 |
| 58 | CDKN2B-AS1 | NR_003529 | 1.3 |
| 59 | APOC1P1 | NR_028412 | -1.6 |
| 60 | LINC00342 | NR_103734 | 1.0 |
| 61 | CYTOR | NR_024204 | 1.0 |
| 62 | SOX2-OT | NR_075091 | -1.6 |
| 63 | LUADT1 | NR_132442 | 1.7 |
| 64 | LINC00143 | NR_033917 | -1.4 |
| 65 | GAS5 | NR_002578 | 1.9 |
| 66 | GHET1 | NR_130107 | 1.2 |
| 67 | GATA6-AS1 | NR_102763 | 1.1 |
| 68 | GAS8-AS1 | NR_122031 | 1.2 |
| 69 | GAPLINC | NR_110429 | -1.3 |
| 70 | FTX | NR_028379 | 1.4 |
| 71 | HAR1A | NR_003244 | 1.0 |
| 72 | HOXA11-AS | NR_002795 | 1.2 |
| 73 | HOTAIRM1 | NR_038366 | 1.1 |
| 74 | HOXA-AS2 | NR_122069 | 1.0 |
| 75 | HAGLR | NR_110458 | 1.0 |
| 76 | HULC | NR_004855 | 1.0 |
| 77 | IRAIN | NR_126453 | 1.0 |
| 78 | HIF1A-AS2 | NR_045406 | 1.5 |
| 79 | HIF1A-AS1 | NR_047116 | 1.7 |
| 80 | KRT7-AS | NR_146274 | 1.0 |
| 81 | KCNQ1OT1 | NR_002728 | 1.0 |
| 82 | CERNA2 | NR_134505 | 1.3 |
| 83 | GAS6-AS1 | NR_044995 | -1.5 |
| 84 | LUCAT1 | NR_103548 | -1.4 |

**
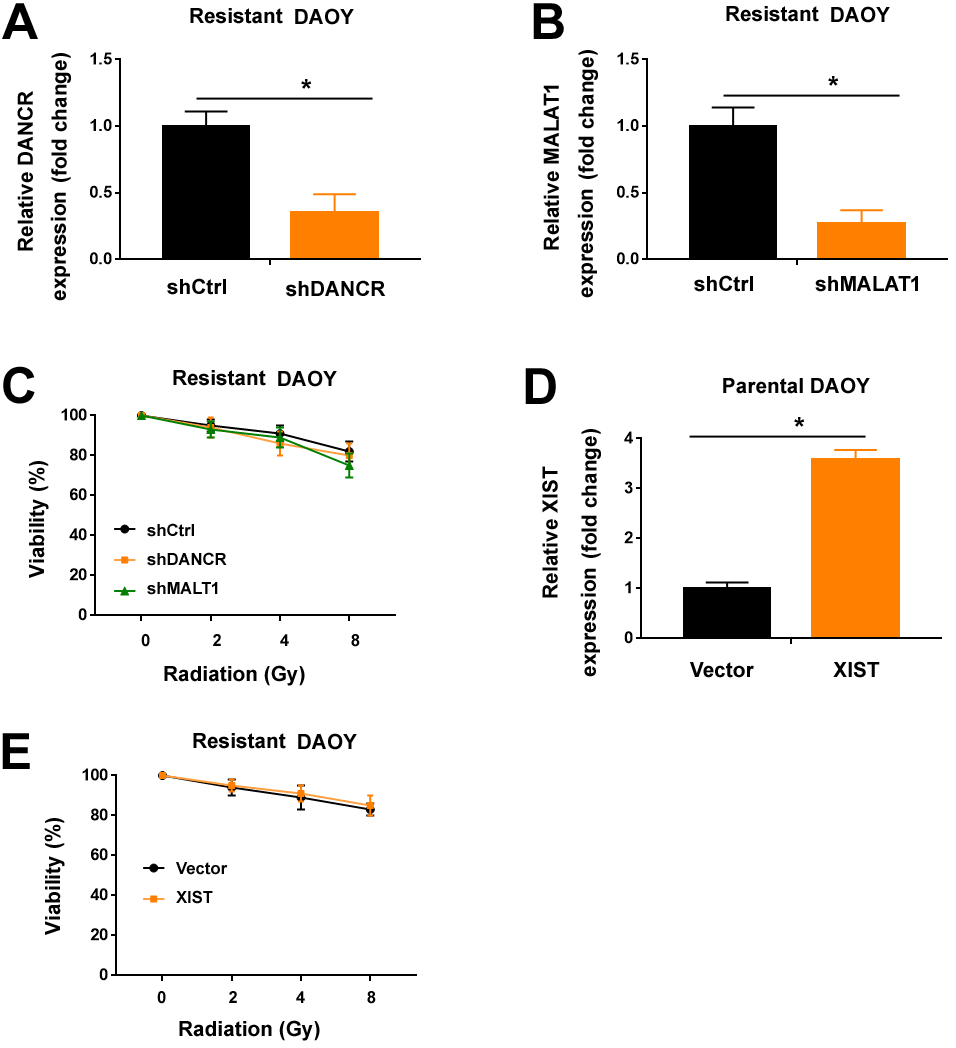
**

**Supplementary Figure S1.** Role of specific lncRNAs in the radiosensitivity of DAOY cells. (A,B) Quantitative real-time PCR analysis of (A) DANCR and (B) MALAT1 abundance in radioresistant DAOY cells transfected with indicated short hairpin RNAs (shRNAs). ^*^*P* < 0.05 vs. Control shRNA (shCtrl). (C) Radioresistant DAOY cells transfected with indicated shRNAs were measured for viability 3 days after radiation. (D) Quantitative real-time PCR analysis of XIST in radioresistant DAOY cells transfected with indicated constructs. ^*^*P* < 0.05 vs. vector. (E) Radioresistant DAOY cells transfected with indicated constructs were exposed to different doses of X-rays and tested for viability after 3 days.

**
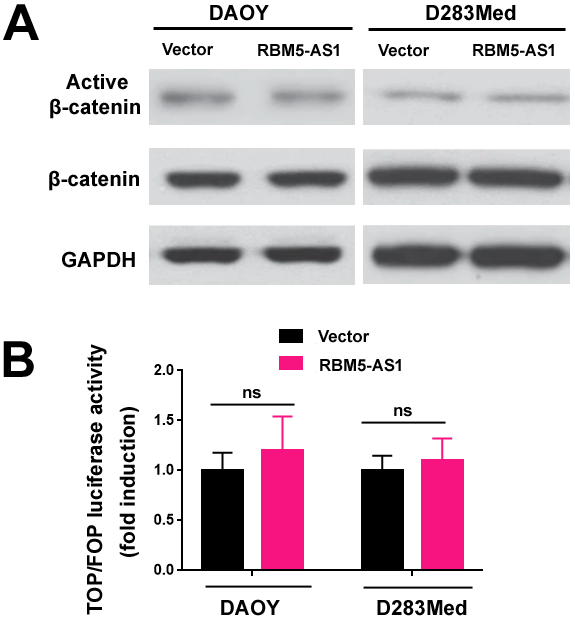
**

**Supplementary Figure S2.** (A) Western blot analysis of non-phospho (active) β-catenin and total β-catenin protein levels. (B) Luciferase reporter assays were performed to assess the effect of RBM5-AS1 overexpression on β-catenin-dependent transcriptional activity. ns indicates no significance.
